# Comparative Analysis of Drying and Preservation Techniques for Whey Powder: Enhancing Physicochemical and Functional Properties

**DOI:** 10.17113/ftb.64.03.26.9455

**Published:** 2026-09-15

**Authors:** Seçil Dede, Sebnem Ozturkoglu-Budak

**Affiliations:** 1 Graduate School of Natural and Applied Sciences, Ankara University, 06110 Dışkapı, Altındağ, Ankara, Turkiye; 2 Department of Dairy Technology, Faculty of Agriculture, Ankara University, 06110 Dışkapı, Altındağ, Ankara, Turkiye

**Keywords:** whey, powder, encapsulation, freeze-drying, spray drying

## Abstract

**Research background:**

Whey is commonly used in powder form in various dairy and food products, including whey-based beverages, yoghurts, sports supplements and bakery items such as cakes and waffles. As a by-product of cheese production, incorporating whey into foods supports both economic and environmental sustainability while enhancing their nutritional and functional qualities, primarily due to its high serum protein content. This study investigates the effects of different technologies, such as spray drying, freeze-drying, and encapsulation combined with freeze-drying, on the functional and nutritional properties of whey. The primary objective is to identify the most effective processing method for preserving the physicochemical and functional integrity of whey.

**Experimental approach:**

Comprehensive analyses were conducted on individually processed and reconstituted whey samples, including the assessment of pH, titratable acidity, dry matter, protein, ash and fat contents, as well as water-holding capacity, rheological behaviour, colour attributes, antioxidant activity and total phenolic content. By comparing the analysis results, the most suitable application was identified based on technological, sensory and functional criteria.

**Results and conclusions:**

Encapsulation combined with freeze-drying was the most effective method, significantly enhancing functional properties. This treatment resulted in the highest antioxidant capacity (1.62 mM Trolox) and water-holding capacity (4.65 %), while largely preserving phenolic compounds (expressed as galllic acid equivalents (GAE), 1.09 mg/mL) and protein content (0.82 %). It also produced the highest consistency index (0.448), indicating improved structural stability. Freeze-drying alone effectively maintained phenolic content (expressed as GAE, 1.13 mg/mL), colour (L*=46.95) and nutritional quality, while improving water-holding capacity (3.91 %) compared to untreated whey. In contrast, spray drying, despite its industrial advantages, resulted in notable reductions in phenolic content (expressed as GAE, 0.82 mg/mL) and antioxidant capacity (0.020 mM Trolox), along with a darker colour (L*=39.5), likely due to thermal degradation. All drying methods increased dry matter content, particularly in encapsulated samples (16.33 %). Overall, encapsulation combined with freeze-drying provided the best balance of functional, nutritional and physicochemical properties, making it a promising approach for enhancing whey stability and shelf life.

**Novelty and scientific contribution:**

These findings highlight the potential of alternative drying and preservation technologies, particularly encapsulation combined with freeze drying, for the sustainable valorisation of whey in functional food applications.

## INTRODUCTION

Whey is the liquid fraction separated from the curd during cheese production. Annually, a substantial amount of whey is generated as a by-product, estimated at approx. 121 million tonnes (*1*). However, fluctuations in global supply mean that a significant portion of whey remains unutilised, contributing to environmental pollution. Whey retains more than 50 % of the total nutrients present in milk (*2*), and due to its high organic content, improper disposal as wastewater poses a serious environmental threat (*3*). Despite these environmental challenges, whey offers considerable nutritional benefits due to its high amount of serum proteins, lactose, vitamins and various minerals (*4*). Therefore, it is essential to explore alternative methods to convert whey into value-added products.

Powdered dairy products have gained increasing popularity worldwide due to their convenience, long shelf life and safety. They are primarily used in manufacturing sectors such as confectionery, infant formula, enteral nutrition formulas and bakery goods, and are extensively produced for export. Their stable characteristics make them well-suited for international trade. U.S. dairy export values rose by 25 %, driven by strong growth in key categories like skimmed milk powder, followed by significant contributions from the EU, New Zealand, Australia and Belarus. The demand for various whey-based ingredients continues to grow, according to the USDA (*5*).

Whey powder can be produced using different drying technologies, such as spray drying and freeze-drying. Freeze-drying helps preserve the physical and chemical properties of the product while ensuring microbial stability. Thus, it gained attention as an alternative drying technology providing improved flavour, aroma and structure retention.

Powdered form is the preferred commercial form due to its long shelf life and ease of handling. Whey drying technologies such as spray drying, freeze-drying, and freeze-drying combined with encapsulation are widely used to improve the stability, handling and functional applicability of whey-based ingredients. These techniques are based on different physicochemical principles that influence moisture removal, particle formation and structural integrity of bioactive components. The most widely used method for producing dairy powder is atmospheric spray drying (ASD), which rapidly evaporates water using high temperature and dry air. This method offers the advantage of a short processing time; however, it can also cause the degradation of bioactive compounds that are sensitive to heat (*6*). Freeze-drying is an alternative drying technique currently in use, which removes moisture through sublimation. It can preserve bioactive components because it operates at low temperatures (around −80 °C). However, this method is time-consuming and costly (*7*). Therefore, a comparative evaluation of these drying approaches is essential to understand how processing conditions govern final product performance and potential food applications.

Encapsulation protects sensitive compounds from environmental factors and allows controlled release. It is an effective method for improving the stability of bioactive compounds by enclosing the core substance within a protective barrier, commonly known as a wall material. Spray drying and freeze-drying are the most widely used methods for producing dry encapsulated powders (*8*). Freeze-drying is particularly suitable for preserving heat-sensitive compounds, as it reduces the risk of degradation caused by high temperatures. However, this method involves high energy consumption and long processing times (*9*). Spray drying, on the other hand, is the preferred method in the food industry due to its adaptability, continuous production capability and cost efficiency. In both spray- and freeze-drying, the encapsulation process involves coating whey with a protective layer. In spray drying, this emulsion is sprayed into a heated chamber, where the solvent quickly evaporates, leaving behind dry particles. Freeze-drying, however, begins with freezing the emulsion and then lowering the pressure so that the frozen solvent transitions directly from solid to gas, creating a porous matrix (*10*).

This study aims to identify the most effective method for preserving the nutritional and functional properties of whey. Antioxidant capacity, total phenolic content, and physicochemical properties, including pH, titratable acidity (percentage of lactic acid), protein content and water-holding capacity of whey powders obtained by spray drying, freeze-drying, and encapsulation followed by freeze-drying were compared.

## MATERIALS AND METHODS

### Materials

The liquid whey used in this study was sweet whey obtained as a by-product of white cheese production (rennet-coagulated cheese) at the Dairy Plant of Ankara University (Ankara, Turkey). Liquid cheese whey is a greenish-yellow, nutrient-rich aqueous byproduct of cheesemaking, comprising 80–90 % of the total milk volume. It consists of approx. 94 % water, lactose, soluble proteins (lactalbumin/lactoglobulin) and minerals. Whey samples obtained from three separate cheese productions conducted at one-week intervals were collected, processed and analysed. The applied processes included spray drying, freeze-drying, and encapsulation followed by freeze-drying to produce powdered cheese whey. Powdered cheese whey is a dried byproduct of cheesemaking produced by removing water from liquid whey using spray drying or freeze-drying. All processing and measurements were performed in triplicate.

### Spray-dried whey powder

Commercially available whey powder produced by atmospheric spray drying was obtained from Enka (Konya, Turkiye). A rotary atomizer operating at 13 500×*g* (GEA Niro atomizer; GEA Process Engineering A/S, Søborg, Denmark) was used for spray drying the samples. The process parameters were set as follows: feed temperature 63 °C, inlet air temperature 180 °C, and outlet air temperature 70 °C. The air flow rate was maintained at 30 000 kg/h, with a drying time of 40-50 s. Whey powders were obtained from three consecutive batches to ensure reproducibility.

### Freeze-drying process

The whey samples were frozen at −80 °C and then freeze-dried for 24 h under a vacuum of 50 Pa and at −58 °C (Alpha 1-2 LDplus; Martin Christ GmbH, Osterode am Harz, Germany). The dried samples were ground and stored at −20 °C until reconstitution.

### Encapsulation process

The freeze-dried samples were also used for the encapsulation treatments. A mixture of 8 % maltodextrin and 2 % gum arabic (both Sigma-Aldrich, Merck, St. Louis, MO, USA) was used as the coating material. The maltodextrin solution was prepared by mixing at 28 °C for 24 h, while gum arabic was mixed at 200 rpm (Unimax 1010 shaker; Heidolph, Schwabach, Germany) and 30 °C for 2 h (*11*). A 1:10 ratio of core to coating material (1 g dry whey powder and 10 g coating material) was used. Encapsulation was carried out for 5 min at 100×*g* using a DIAX 900 UltraTurrax homogenizer (Heidolph). Dried capsules were obtained after a second freeze-drying process for 48 h under the same conditions.

### Reconstitution process

Whey powders obtained by spray drying, freeze-drying, and encapsulation followed by freeze-drying were reconstituted with sterile distilled water to obtain solutions containing 10 % (*m*/*V*) total solids. This standardisation was applied to ensure a comparable testing matrix across all treatments, based on the solid-not-fat (SNF) content of the bovine milk from which each whey sample was derived.

### Physicochemical analyses

Physicochemical properties of untreated whey and reconstituted whey powder samples were analysed according to the methods described below. The pH was measured by direct insertion of a pH meter (Starter300; Ohaus, Shanghai, PR China). Titratable acidity (expressed as percentage of lactic acid) was determined according to the AOAC method (*12*). Dry matter content was analysed using the gravimetric method at 102 °C (*12*). Fat content and total nitrogen (TN) content were determined using the Gerber-Van Gulik method and the Kjeldahl method, respectively (*12*). Nitrogen content was converted to protein content by applying a conversion factor of 6.38. Ash content was obtained by incinerating the sample in a muffle furnace (LE 6/11; Nabertherm GmbH, Lilienthal, Germany) at 550 °C for 6 h. The water-holding capacity (WHC) of the samples (25 g) was determined using the centrifugation method described by Isanga and Zhang (*13*). Samples were centrifuged at 8000×*g* for 15 min at 4 °C using a Sigma 3–18K centrifuge (Sartorius AG, Göttingen, Germany). WHC (in %) was calculated using the following equation:


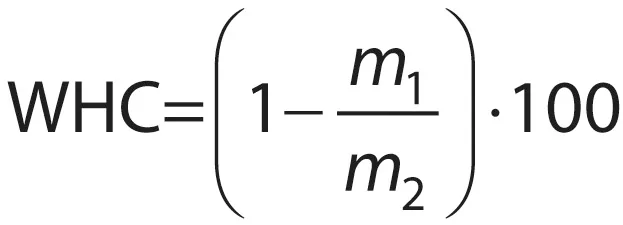
 /1/

where *m*_1_ is the mass of the supernatant obtained after centrifugation, and *m*_2_ is the initial mass of the sample.

### Rheological analyses

Rheological analyses were performed using a Kinexus Pro+ rheometer (Malvern Panalytical, Malvern, UK) with a 40-mm stainless steel 4° conical geometry spindle and a 1-mm gap. The temperature for the frequency sweep measurements was set at 5 °C. The shear rate was set in the range of 0.1–300 s^-1^. The flow curves were fitted to the power law model using non-linear regression. The power law model equation was as follows:


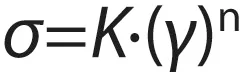
 /2/

where *σ* is the shear stress (Pa), *K* is the consistency index (Pa⋅s^n^), *γ̇* is the shear rate (s^−1^), and *n* is the flow behaviour index (dimensionless).

### Colour determination

Colour measurements were carried out using a portable colour spectrophotometer (model SL400; Ser-Lab, İstanbul, Turkiye) based on the CIE L*a*b* colour space. After calibration with white and black backgrounds, samples were individually placed in the container, and measurements were recorded for L* (lightness), a* (red-green) and b* (yellow-blue) values.

### Determination of total phenolic content

Total phenolic content in whey and reconstituted whey samples was determined using the Folin-Ciocalteu method. A sample volume of 5 mL was mixed with 25 mL of a *V*(ethanol):*V*(acetic acid):*V*(water)=50:42:8) mixture (Sigma-Aldrich, Merck) and centrifuged at 9000×*g* (Sartorius AG) for 15 min. The clear supernatant obtained was used as the extract for total phenolic analysis. For the analyses, 100 µL of the extract, 500 µL of Folin-Ciocalteu reagent (Merck KGaA, Darmstadt, Germany), 1.5 mL of 20 % Na_2_CO_3_ and 7.9 mL of distilled water were mixed in a test tube. The mixtures were vortexed and incubated in the dark at room temperature for 1 h. After incubation, absorbance was measured in triplicate at 760 nm using a UV-Vis spectrophotometer (Lambda 25; PerkinElmer, Waltham, MA, USA). Calculations were done using a gallic acid standard curve. For this purpose, 5 mg/mL gallic acid (Sigma-Aldrich, Merck) was prepared as the stock solution, and working solutions were prepared at concentrations of 0.05–4 mg/mL. Results were expressed as GAE in mg/mL (R^2^=0.97).

### Determination of antioxidant activity

The antioxidant activities of whey and reconstituted whey samples were determined by the 2,2-diphenyl-1-picrylhydrazyl (DPPH) free radical scavenging method. A volume of 100 µL of the extracted sample was added to 2 mL of DPPH solution (0.2 mM DPPH in 95 % methanol; Sigma-Aldrich, Merck) and kept in the dark for 30 min at 4 °C. At the end of the incubation, absorbance was measured at 517 nm using a UV-Vis spectrophotometer (Lambda 25; PerkinElmer). Distilled water was used as a blank. The inhibition percentage was calculated using the following equation:


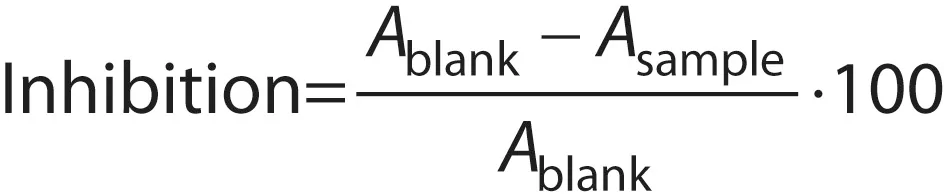
 /3/

where *A*_blank_ is the absorbance of the DPPH solution with water instead of the sample, and *A*_sample_ is the absorbance of the DPPH solution with the sample. Each value was compared with Trolox standards prepared at concentrations of 0.05–5.00 mM. The measured absorbances were converted to μM Trolox equivalents (TE).

DPPH radical scavenging activity, representing antioxidant activity, was expressed as mM Trolox equivalents TE) using a Trolox standard curve prepared with concentrations ranging from 0.05 to 2.50 mM (R^2^=0.99).

### Statistical analyses

Statistical analyses were carried out using Minitab v. 17 (*14*). Analysis of variance (ANOVA) was performed to determine statistical differences among samples (p<0.05). Tukey's *post-hoc* multiple comparison test was applied to identify which specific treatments caused the differences.

## RESULTS AND DISCUSSION

### pH value and physicochemical properties

The pH values and other chemical properties of all whey samples are shown in Table 1. The pH values were similar across all treatment groups (6.49–6.64), with no statistically significant differences (p>0.05). This indicates that the different drying methods did not affect the buffering capacity of whey and that pH remained stable. However, titratable acidity was significantly higher in the encapsulated samples (0.16 %) (p<0.05). This increase can be attributed to the acidic contributions of coating materials such as maltodextrin and gum arabic, as well as the more controlled release of organic acids within the microencapsulated structures (*15*). Encapsulation has been shown to effectively preserve the acidic profile of whey and may even increase the concentration of its acidic components (*16*). In contrast, spray dried samples exhibited low acidity (0.09 %), comparable to untreated whey, indicating organic acid loss due to heat exposure during drying.

**Table 1:** Chemical properties of whey with different treatments

Property	Untreated whey	Freeze-dried RS whey	Encapsulation+freeze-dried RS whey	Spray-dried RS whey
pH	(6.49±0.02)^A^	(6.50±0.02)^A^	(6.50±0.04)^A^	(6.64±0.01)^A^
TA/%	(0.092±0.003)^B^	(0.103±0.003)^B^	(0.161±0.005)^A^	(0.090±0.001)^B^
*w*(dry matter)/%	(6.36±0.02)^D^	(8.32±0.05)^C^	(16.33±0.06)^A^	(8.72±0.03)^B^
*w*(protein)/%	(0.87±0.01)^A^	(0.81±0.05)^AB^	(0.82±0.26)^AB^	(0.76±0.04)^B^
*w*(ash)/%	(0.430±0.000)^B^	(0.64±0.02)^A^	(0.64±0.02)^A^	(0.67±0.02)^A^
*w*(fat)/%	(0.401±0.002)^B^	(0.403±0.000)	(0.402±0.000)	(0.400±0.000)
WHC/%	(2.72±0.04)^C^	(3.912±0.005)^B^	(4.65±0.04)^A^	(4.60±0.16)^A^

RS=reconstituted, TA=titratable acidity (lactic acid percentage), WHC=water-holding capacity. Differences between all samples (p<0.05) are indicated by capital letters in superscript. Values are shown as mean±S.D.

Although all samples were reconstituted to a standardised 10 % (*m*/*V*) total solids, the measured dry matter content varied among treatments because of differences in powder composition and matrix structure. Freeze-dried and spray-dried whey powders showed lower dry matter mass fractions (8.32 and 8.72 %, respectively), which may be attributed to their higher solubility and more complete rehydration, resulting in a more homogeneous dispersion of solids in the aqueous phase. In contrast, the encapsulation-assisted freeze-dried sample exhibited a significantly higher dry matter mass fraction (16.33 %), which can be explained by the presence of carrier materials used during encapsulation. These carriers increase the total solid fraction and may not fully dissolve or disperse during reconstitution, thereby contributing to higher measured dry matter values. Therefore, the observed differences reflect variations in powder composition and rehydration behaviour rather than inconsistencies in the reconstitution protocol. Dry matter content also correlated positively with water-holding capacity.

The protein mass fraction was highest in the untreated whey (0.87 %) but decreased to as low as 0.76 % in the spray-dried samples. This reduction is associated with protein denaturation caused by high temperatures, which affect the secondary and tertiary structures of proteins. Freeze-drying and encapsulation, being carried out at lower temperatures or in protective environments, resulted in more limited protein loss (*17*). Ghanimah *et al*. (*18*) also reported that higher protein mass fractions led to increased functionality. Farahmandpour *et al.* (*19*) investigated the effects of whey protein supplementation on inflammatory markers and oxidative stress, highlighting that protein processing methods can influence nutritional value. They reported that proteins processed at lower temperatures retained more functional properties than those exposed to higher temperatures. The study also emphasised that encapsulation helps preserve the biochemical integrity of proteins by preventing denaturation. The reduction in protein content also had an indirect effect on the phenolic compound content and antioxidant activity of whey, as protein–phenolic interactions can influence antioxidant stability (*20*).

Ash mass fraction was lowest in the untreated whey (0.43 %), while significantly higher values were observed in all processed samples (0.64–0.67 %) (p<0.05). This increase can be explained by water removal during drying, which leads to a concentration of minerals (*21*). However, despite the increase in ash mass fraction, there was a tendency for phenolic compound concentration to decrease. This suggests that while thermal processing helps retain mineral content, it may cause degradation of heat-sensitive phenolic compounds.

No significant differences were observed among the samples with respect to fat content (0.40 %). These findings indicate that whey fats are stable under the applied thermal and mechanical treatments and are not markedly influenced by the processing method.

Water-holding capacity increased significantly, particularly in the encapsulated samples (4.65 %). This enhancement is attributed to the hydrophilic nature of coating agents such as maltodextrin and gum arabic. Spray-dried whey exhibited a similarly high water-holding capacity (4.60 %); however, this capacity is associated more with the presence of carrier materials (8 % maltodextrin and 2 % gum arabic) than with protein content. Freeze-dried whey, on the other hand, showed a lower water-holding capacity (3.91 %). These differences are directly related to variations in dry matter and protein content, indicating that the processing technique substantially influences the microstructure and water-binding capacity of the product. Farahmandpour *et al.* (*19*) also reported that the water retention capacity of whey products subjected to different heat treatments and additives ranged from 3.75 to 4.70 %, which is consistent with the present findings and demonstrates that encapsulation and spray drying processes enhance the physical properties of whey. Encapsulation has been shown to increase the capacity to bind water molecules.

### Rheological properties

As shown in Table 2, significant differences were observed among the samples in terms of consistency index (*K*) and flow behaviour index (*n*). Encapsulated and then freeze-dried whey exhibited the highest consistency index (0.448 Pa·s), followed by spray-dried (0.190 Pa·s) and freeze-dried (0.130 Pa·s) whey. The untreated sample displayed the lowest value (0.020 Pa·s). Similarly, McEntee *et al.* (*22*) determined strong gelation properties in reconstituted powders (*w*(protein)=5 %) following heat treatment at 90 °C. The elevated consistency is directly linked to increased dry matter content, enhanced water retention, and the dense nature of the encapsulated matrix. Regarding flow behaviour, spray-dried whey showed a flow behaviour index closest to Newtonian behaviour (*n*=0.696), while both encapsulated and freeze-dried samples exhibited lower *n* values, indicating pseudoplastic behaviour. This suggests that the method of whey processing has a significant effect on the physical properties of the final product. Treatments such as freeze-drying, encapsulation and spray drying enhance the consistency of reconstituted whey, thereby influencing its flow and viscosity.

**Table 2:** Rheological values of whey with different treatments

Index	Untreated whey	Freeze-dried RS whey	Encapsulation+freeze-dried RS whey	Spray-dried RS whey
*K*	(0.020±0.001)^D^	(0.130±0.000)^C^	(0.448±0.002)^A^	(0.190±0.001)^B^
*n*	(0.560±0.009)^B^	(0.247±0.000)^C^	(0.209±0.003)^D^	(0.696±0.006)^A^

RS=reconstituted, *K*=consistency index, *n*=flow behaviour index. Differences between all samples (p<0.05) are indicated by a capital letter in superscript. Values are shown as mean±S.D.

Onay-Ucar *et al.* (*23*) examined the rheological properties of whey and reported that the consistency index (*K*) increased to 0.45 % with encapsulation, compared to 0.025 % in untreated samples. Flow behaviour index (*n*) was highest in spray-dried reconstituted whey, indicating increased flowability compared to freze-dried, and encapsulated and then freze-dried reconstituted whey. Similarly, Gausemel *et al.* (*24*) found that the flow behaviour index (*n*) of spray-dried whey powder processed at low temperatures demonstrated better flowability. In another study, Zhou *et al*. (*25*) evaluated the flowability of spray- and freeze-dried milk powders and reported that spray-dried milk powders generally exhibit superior flowability compared to freeze-dried powders. This difference is primarily due to the formation of small, spherical, dense particles during spray drying, which reduces interparticle friction. In contrast, freeze-drying often results in porous, irregular, sheet-like, or larger, more cohesive particles that hinder flow.

### Colour properties

The L*, a* and b* values obtained from colour analysis (Table 3) indicate that processing methods significantly influence the colour profile of whey. Encapsulated whey showed the highest lightness value (L*=55.7), while spray-dried whey exhibited the lowest (L*=39.5), corresponding to a darker colour. These differences can be attributed to Maillard reactions, polyphenol oxidation and variations in particle size. In addition, caramelisation and browning reactions likely occurred during spray drying as a result of the high processing temperatures. These findings suggest that the whey processing method has a significant impact on its colour properties. Encapsulation produces a lighter, more yellow product, while the commercial form appears darker and more neutral, reflecting the direct effects of additives or processing techniques.

**Table 3:** Colour values of whey with different treatments

Parameter	Untreated whey	Freeze-dried RS whey	Encapsulation+freeze-dried RS whey	Spray-dried RS whey
L*	(46.8±1.6)^B^	(47.0±0.2)^B^	(55.7±0.4)^A^	(39.50±0.00)^C^
a*	(−2.05±0.05)^A^	(−2.32±0.00)^B^	(−2.95±0.05) ^C^	(−2.80±0.00)^C^
b*	(0.2±0.4)^A^	(0.3±0.4)^A^	(1.6±1.0)^A^	(1.70±0.00)^A^

RS=reconstituted. Differences between all samples (p<0.05) are indicated by a capital letter in superscript. Values are shown as mean±S.D.

Başyiğit and Karaaslan (*26*) compared the colour characteristics of wild mushrooms dried by freeze-drying, vacuum-drying, cabinet-drying, and solar-energy drying. The highest L* value (58.15) was observed with the freeze-drying method, while the lowest (42.15) occurred with solar-energy drying. These differences were attributed to enzymatic browning resulting from varying oxygen levels. Overall, freeze-drying was identified as the most effective technique for preserving colour characteristics.

In spray drying, discolouration may occur due to high inlet air temperatures or prolonged exposure of the powder in the dryer, which can lower L* values (*27*). This effect has also been attributed to the concentration of low-moisture samples at very high temperatures and to Maillard reactions induced by heat. For both spray- and freeze-drying techniques, the a* parameter showed low positive values, indicating a reddish hue, while the b* parameter showed low positive values, indicating a yellow hue. The a* and b* values of the lyophilised powder were 7.39 and 13.23, respectively, while the spray-dried powder showed values of 6.86 and 16.16, corresponding to lower redness.

### Phenolic content

As shown in Table 4, the total phenolic content was similar in the untreated and freeze-dried whey samples, expressed as galllic acid equivalents (GAE) (1.15 and 1.13 mg/mL, respectively), but decreased significantly in the spray-dried samples (0.82 mg/mL). In contrast, the encapsulated whey exhibited an intermediate phenolic content (1.09 mg/mL). These losses are primarily attributed to degradation caused by thermal processing. Because high temperatures can lead to the degradation of phenolic compounds, freeze-drying at low temperatures preserves most of these compounds (*28*). Consequently, the phenolic content of freeze-dried whey remained similar to that of untreated whey. Encapsulation, on the other hand, can help preserve active compounds while altering their bioavailability. Studies have shown that although encapsulation can increase the bioavailability of phenolic compounds, it may also prevent some compounds from existing in free form, resulting in a lower measured phenolic content (*29*). In addition, unlike the other samples, the whey obtained by encapsulation contains a coating material, which likely contributes to the lower measured phenolic content in the analysed sample. Due to the high temperatures involved, the spray drying may cause the degradation of heat-sensitive phenolic compounds, while also facilitating temperature-related Maillard reactions that can further contribute to phenolic compound loss (*30*). Onay-Ucar *et al.* (*23*) reported the phenolic content of original whey as 1.20 mg/mL and that of its freeze-dried form as 1.15 mg/mL. In spray-dried whey samples, the phenolic content decreased to 0.78 mg/mL. Li *et al.* (*31*) evaluated the interaction between whey protein and polyphenolic compounds and reported that phenolic content could reach up to 1.12 mg/mL using encapsulation. Furthermore, this value was maintained between 1.10 and 1.15 mg/mL when low-temperature lyophilisation was applied. In contrast, Farahmandpour *et al.* (*19*) reported that high-temperature treatments led to greater loss of phenolic compounds, reducing the content to 0.85 mg/mL in whey processed by spray drying.

**Table 4:** Functional properties of whey with different treatments

Property	Untreated whey	Freeze-dried RS whey	Encapsulation+freeze-dried RS whey	Spray-dried RS whey
*γ*(phenolics as GAE)/(mg/mL)	(1.15±0.02)^A^	(1.10±0.1)^A^	(1.09±0.01)^B^	(0.82±0.01)^C^
Antioxidant capacity as *c*(TE)/mM	(1.30±0.02)^B^	(1.40±0.01)^B^	(1.60±0.02)^A^	(0.58±0.02)^C^

RS=reconstituted, GAE=gallic acid equivalent, TE=Trolox equivalent. Differences between all samples (p<0.05) are indicated by a capital letter in superscript. Values are shown as mean±S.D.

### Antioxidant activity

Total antioxidant activity, expressed as Trolox equivalents, although closely associated with phenolic content, was highest in the encapsulated whey (1.62 mM). This suggests that encapsulation can effectively protect bioactive compounds from oxidation and enhance their bioavailability. In contrast, the antioxidant capacity of the spray-dried sample decreased to 0.58 mM Trolox, indicating that high processing temperatures led to structural degradation of antioxidant compounds.

Freeze-dried whey exhibited moderate antioxidant activity, with a value of 1.40 mM Trolox, while untreated whey showed a similar concentrations of 1.30 mM Trolox. Although antioxidant capacity is partially preserved in both forms, these values were lower than those observed in the encapsulated whey sample. Freeze-drying, performed at low temperatures under vacuum, minimises thermal damage to antioxidant components. In contrast, powdered whey produced by spray drying showed the lowest antioxidant activity at 0.58 mM Trolox, suggesting that high-temperature treatments generally result in the loss of antioxidant compounds. The elevated temperatures (180 °C) used during spray drying can cause oxidation of these components, reducing their functional properties (*30*).

As a result, the whey processing method had a significant impact on antioxidant content. Encapsulation enhanced the preservation of antioxidant compounds, while spray drying generally led to their loss. Onay-Ucar *et al.* (*23*) examined the antioxidant capacity of different whey protein fractions and reported values ranging from 1.2 to 1.5 mM Trolox. These values are consistent with the antioxidant capacities measured in this study for untreated whey (1.30 mM Trolox) and lyophilised whey (1.40 mM Trolox). The ability of lyophilisation to preserve antioxidant activity is attributed to its low-temperature process, which minimises the degradation of phenolic and other bioactive compounds. However, findings from Onay-Ucar *et al.* (*23*) also indicate that high-temperature methods, such as spray drying, can reduce antioxidant capacity.

## CONCLUSIONS

In this study, the physical, chemical and functional properties of whey samples processed by three different techniques, spray drying, freeze-drying and encapsulation, were comparatively evaluated. The results indicated that these methods differently influenced the structural integrity and functional quality of whey.

Encapsulation combined with freeze-drying was found to be the most effective method for preserving the nutritional value and functional properties of whey. This technique yielded the highest dry matter content (16.33 %), water-holding capacity (4.65 %), consistency index (0.448), and antioxidant activity (1.62 mM Trolox). In addition, it maintained relatively high phenolic content expressed as gallic acid equivalents (GAE) (1.09/mL) and protein levels (0.82 %), indicating effective protection of bioactive compounds. These results suggest that carrier materials, such as maltodextrin and gum arabic, enhance the structural stability and protect bioactive compounds. Freeze-drying alone also proved to be suitable for heat-sensitive components, preserving phenolic content, expressed as GAE (1.13 mg/mL), antioxidant capacity (1.40 mM Trolox) and protein content (0.81 %), while maintaining colour (L*=46.95) and improving water-holding capacity (3.91 %) compared to untreated whey. However, its effectiveness in improving consistency (0.130) and water-holding capacity remained lower than that of encapsulation. In contrast, spray drying, despite its operational advantages, resulted in notable reductions in protein content (0.76 %), phenolic compounds, expressed as GAE (0.82 mg/mL), and antioxidant activity (0.58 mM Trolox), along with decreased lightness (L*=39.5), indicating quality deterioration due to high processing temperatures. Therefore, although spray drying offers economic and practical benefits, its application should be carefully optimised, particularly for products requiring the preservation of heat-sensitive bioactive components.

In conclusion, although encapsulation emerges as the preferred method for enhancing the functional and nutritional properties of whey, the choice of processing technique should depend on the intended application of the product, such as a functional food additive, texture enhancer or bioactive carrier. The advantages and limitations of each method should be carefully considered within a comprehensive evaluation framework.
